# Pathway analysis and transcriptomics improve protein identification by shotgun proteomics from samples comprising small number of cells - a benchmarking study

**DOI:** 10.1186/1471-2164-15-S9-S1

**Published:** 2014-12-08

**Authors:** Jing Sun, Guang Lan Zhang, Siyang Li, Alexander R Ivanov, David Fenyo, Frederique Lisacek, Shashi K Murthy, Barry L Karger, Vladimir Brusic

**Affiliations:** 1 Cancer Vaccine Center, Dana-Farber Cancer Institute, Boston, MA, USA; 2 Department of Computer Science, Metropolitan College, Boston University, Boston, MA, USA; 3 Barnett Institute, Northeastern University, Boston, MA, USA; 4 Laboratory of Computational Proteomics, Center for Health Informatics and Bioinformatics, New York University Medical Center, New York, NY, USA; 5 Proteome Informatics Group, Swiss Institute for Bioinformatics, CMU, Geneva, Switzerland; 6 Department of Chemical Engineering, Northeastern University, Boston, MA, USA; 7 School of Science and Technology, Nazarbayev University, Astana, Kazakhstan

## Abstract

**Background:**

Proteomics research is enabled with the high-throughput technologies, but our ability to identify expressed proteome is limited in small samples. The coverage and consistency of proteome expression are critical problems in proteomics. Here, we propose pathway analysis and combination of microproteomics and transcriptomics analyses to improve mass-spectrometry protein identification from small size samples.

**Results:**

Multiple proteomics runs using MCF-7 cell line detected 4,957 expressed proteins. About 80% of expressed proteins were present in MCF-7 transcripts data; highly expressed transcripts are more likely to have expressed proteins. Approximately 1,000 proteins were detected in each run of the small sample proteomics. These proteins were mapped to gene symbols and compared with gene sets representing canonical pathways, more than 4,000 genes were extracted from the enriched gene sets. The identified canonical pathways were largely overlapping between individual runs. Of identified pathways 182 were shared between three individual small sample runs.

**Conclusions:**

Current technologies enable us to directly detect 10% of expressed proteomes from small sample comprising as few as 50 cells. We used knowledge-based approaches to elucidate the missing proteome that can be verified by targeted proteomics. This knowledge-based approach includes pathway analysis and combination of gene expression and protein expression data for target prioritization. Genes present in both the enriched gene sets (canonical pathways collection) and in small sample proteomics data correspond to approximately 50% of expressed proteomes in larger sample proteomics data. In addition, 90% of targets from canonical pathways were estimated to be expressed. The comparison of proteomics and transcriptomics data, suggests that highly expressed transcripts have high probability of protein expression. However, approximately 10% of expressed proteins could not be matched with the expressed transcripts.

## Background

Proteomics is the large-scale study of proteins expressed in an organism, tissues, or cells.

Protein expression changes over time in response to various stimuli or to change of conditions. Cellular proteome is a set of proteins expressed in a specific cell type, across various subcellular locations, whereas human proteome is a set of proteins encoded by some 25,000 protein-coding genes of human genome [1]. High-throughput sequencing has shown that more than 95% of human protein coding genes produce splice variant transcripts [2]. More than 260,000 protein variants resulting from alternative splicing have been annotated to date [3]. A wide variety of post-translational modifications (PTM) occur in proteins, often changing their structure and function. PTMs include phosphorylation, ubiquitination, glycosylation, methylation, acetylation, sulfation, oxidation, and nitrosylation, among many others [4]. A widely accepted estimate is that more than 2 million protein variants make the posttranslated human proteome in any human individual [5]. This estimate excludes natural recombinant proteins such as T cell receptors and antibodies and the majority of PTMs.

### High-throughput technologies

Molecular profiling of samples representing healthy or diseased states, pre- and postintervention, or time series during disease progression is important research topic in biomedicine. Such profiling supports the discovery and evaluation of cellular-level pathways of disease progression, characterization of biomarkers, identification of therapeutic targets, and their applications for improved diagnosis, prognosis, monitoring, and selection of therapies. This quest is supported by the emerging technologies of genomics, transcriptomics, and proteomics. Genomics technologies help identify variation of human genome and its role in disease associations, but they do not provide information about availability of transcripts (RNA expression). Transcriptomics studies the RNA transcribed from a particular genome under various conditions. Transcriptomic studies link the analysis of genome and proteome because they provide critical information about gene regulation and also about availability of mRNA for protein translation. Principal strengths of genomics and transcriptomics lay in the ability to amplify genetic material for extraction of genetic and transcript profiles even from a single cell [6]. On the contrary, proteomics profiling requires utilization of larger numbers of cells. Identification of proteome from a single cell is currently available for the analysis of cell lines [7] as it allows for a very limited proteome analysis [8]. Currently a single cell proteome analysis is not a viable option for clinical applications. Larger samples yield better proteome coverage, while smaller samples yield progressively smaller coverage of the expressed proteome. On the other hand, transcriptome analysis provides limited information: patterns of changes in gene expression do not necessarily correlate well with patterns of changes in protein expression [9]. The limited correlation of transcript and protein expression is particularly notable in the study of human clinical samples. The reasons for such discrepancies include errors in measurement, noise in regulation of gene expression, presence of posttranslational modifications, variation in gene-specific regulation of translation, and varying dynamics of protein degradation under different conditions [10]. Furthermore, genomic and transcriptomic studies cannot provide information on PTMs, and quantity of proteins. About a third of transcripts, although expressed, do not get translated into proteins [11] and the lifetime of proteins can differ by orders of magnitude even for proteins that have similar translation rates [9]. Because of their limitations, genomics, transcriptomics, and proteomics complement each other - each of them provides a valuable but incomplete insight into molecular profiles that characterize healthy and diseased states represented by the studied sample.

To achieve clinical goals of proteomics, we need to target samples that often comprise extremely low numbers of cells. These include, among others, circulating tumor cells [12,13], circulating endothelial cells [14-16], samples collected using fine needle aspirates [17], and samples collected by laser capture microdissection [18]. Limited numbers of cells are available from samples that contain mixed normal and transformed (tumor) cells, which is a particular problem in tissue samples with early stage cancer [19]. The latest microproteomics methods enable proteome profiling from tiny samples (less than 1,000 cells) and are thus suitable for moving the frontier of clinical applications. Such proteome studies can yield a few thousand proteins [20,21], which was confirmed in this report. Deep proteomics can yield more than 10,000 proteins from cell line samples [7], but the number of proteins identifiable from clinical samples is usually much smaller.

### Technological advances

Significant improvements in instrumentation (sensitivity, throughput, resolution of separation) [9], sample processing [11], and bioinformatics [22,23] help comprehensive proteome profiling. Nevertheless, proteome profiling suffers from problems of incomplete data coverage and inconsistencies between individual runs [24]. While this problem is less pronounced in protein identification using large samples derived from cell lines where vast majority of proteins are expressed ubiquitously [11,25] this problem is pronounced when small samples are used. Goh *et al*. [24] have argued that biological networks analysis provides robust models and interpretations that increase coverage while the analysis of protein interaction groups and biological pathways help improve coverage of proteins and complement quantitative proteomics data. They defined biological networks as groups of genes or proteins that are linked through a shared set of functional relationships and pathways as well-described biological networks involved in metabolic and regulatory processes. Several methods for utilization of biological networks for improvement of identification (coverage) have been described, including analysis of overlaps, clique enrichment analysis, proteomics expansion pipeline, Maxlink, and shortest-path methods (reviewed in [24]). The improvement of inconsistencies between different runs can be pursued using overlap analysis, direct group analysis, and network-based analysis (reviewed in [24]). Gene expression profiles and pathway analysis can be used to define candidates for targeted proteomics [26] discovery and improve identification sensitivity of expressed proteins. Targeted proteomics is more sensitive than unbiased screening - the sensitivity of specific protein identification using targeted proteomics can be an order of magnitude larger than sensitivity of unbiased screening [27].

Here, we compared newly measured MCF-7 proteomics data from several small-size samples. The detected proteins were mapped to standardized gene names. The target proteins were predicted to be present in samples using enriched gene sets among canonical pathways collections in MSigDB [28]. In addition, the expanded gene sets were compared to the transcriptomics data extracted from the literature. We benchmarked the consistency and coverage of proteomes identified in different small-sample runs and defined a strategy for proteome profiling and quantitation using the analysis of expressed canonical pathways.

## Methods

**Figure 1:**
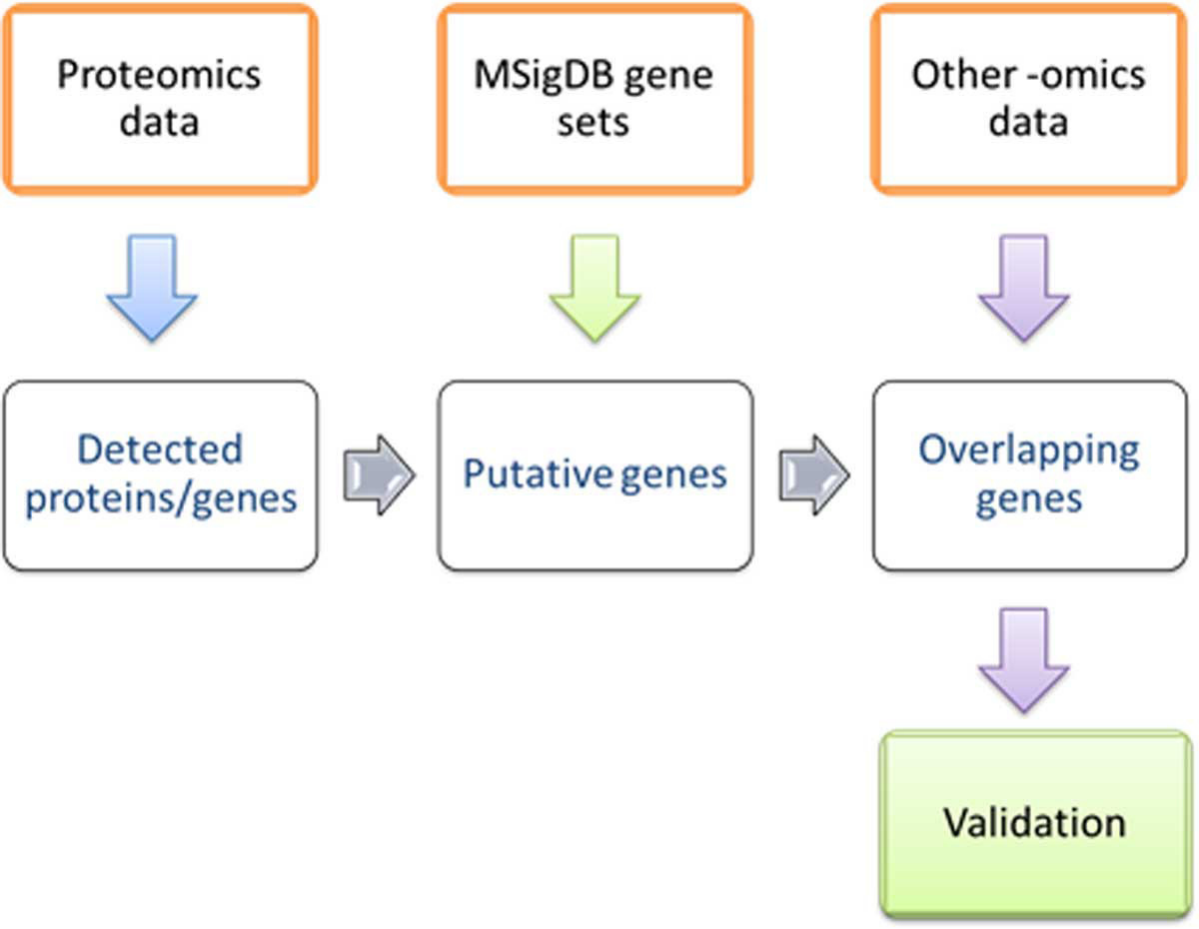
**The overall design of the study**. This study used three data sets: proteomics data, gene sets from MSigDB, and gene expression data. Proteomics data sets utilized data from small samples. This study focused on developing methods for elucidation of proteins that are present in actual samples from small sample data.

The overall design of this study is shown in Figure 1. Small sample, as few as 50-cell, and larger sample (500, 1000, 2500, and 5000-cell) proteomics data from MCF-7, an estrogen receptor positive (ER+) breast cancer cell line (ATCC number: HTB-22) [29] were compared to gene sets from MSigDB and to transcriptomics data. The enrichment of detected targets among gene sets from canonical pathways (CP collection in MSigDB) collection were calculated. The genes from enriched gene sets were used for identification and assessment of possible protein expression in the sample. The detected targets and their enriched gene sets were compared to MCF-7 transcriptomics data. The initial validation was performed by comparison to the partial proteome estimated to cover 50% of expressed proteins in runs comprising as much as 5,000 cells.

### Data set acquisition

#### Proteomics data

10^7 ^of MCF-7 human breast adenocarcinoma cells (ATCC, Manassas, VA) were rinsed twice with 1 mL volumes of DPBS and lysed with sonication on ice in 8 M urea, 2 M thiourea, 5 mM TCEP in 25 mM ammonium bicarbonate (ABC), pH 8.4 for 15 min. Extracted proteins were processed using a previously described protocol[30]. In brief, the extract was reduced in 5 mM TCEP for 30 min at room temperature and alkylated in 20 mM iodoacetamide (IAA) for 30 min in the darkness. Prior to digestion, the resulting lysate was diluted 10-fold with 25 mM ABC, pH 8.4 to bring the concentrations of urea and thiourea to 0.8 M and 0.2 M, respectively. Protein digestion was performed with endoproteinase Lys-C (sequencing grade, Promega, Madison, WI) for 4h at an enzyme/substrate (E:S) of approximately 1:50 and followed by an addition of sequencing grade trypsin (Promega) at an E:S ratio of approximately 1:50 and overnight digestion in a shaker at 37 °C. The total volume of the resulting digest was 1000 µL.

The monolithic microSPE and analytical porous layer open tubular (PLOT) columns were polymerized as described in [31]. For LC separation, 5 cm of 50 μm i.d. PS-DVB monolithic SPE precolumn was connected with a 4.2 m PLOT using a PicoClear Tee (NewObjective, Woburn MA). Digested lysates were first loaded on the monolithic SPE precolumn at a flow rate of 200 nL/min using a NCS 3500 RS pump (Dionex, Sunnyvale, CA). Then, entrapped and desalted digests were eluted off the precolumn and separated on the PLOT column using a linear solvent gradient at a 20 nL/min flow rate. The separation was performed using a 4-hour gradient of 0%-27% mobile phase B (mobile phase A: 0.1% FA in water; mobile phase B, 0.1% FA in ACN). Nano ESI spray was enabled using an electrospray voltage of 1.1 kV and a distal coated tip (NewObjective) butt-to-butt connected with an outlet of the PLOT column via a zero dead volume PicoClear union (New Objective). Ion transfer tube temperature was set for 275 ^0^C.

MS detection was performed using a top 12 MS/MS data-dependent scans on the Q Exactive (Thermo Fisher Scientific) mass spectrometer. Full MS scans were acquired over the range of m/z 380-1600 Th with resolution set to 70,000 and an automatic gain control (AGC) target set to 3x10^6^. The 12 most intense parent ions excluding singly charged ions and ions with unassigned charges were selected for higer-energy collisional dissociation (HCD) fragmentation with a normalized collision energy (NCE) set to 28%. The MS/MS spectra were analyzed in the Orbitrap mass analyzer using resolution set to 17,500 and AGC set to 1x10^5^. The isolation window was set to 2 m/z and dynamic exclusion was set to 60 s. The maximum ion injection time was set to 20 ms for full MS scans and 120 ms for MS/MS.

LC-MS/MS raw data files were analyzed using Proteome Discoverer 1.4 (Thermo Fisher Scientific) by two search engines Sequest HT (Thermo) and Mascot (Matrix Science) against the UniProt human database (2013 Jan version, containing 139905 sequences).

Carbamidomethylation (57.021 Da was set as a fixed modification and N-terminal acetylation, methionine oxidation and deamidation (NQ) were set as variable modifications. The precursor peptide mass tolerance was set to 10 ppm and fragment tolerance to 0.05 Da. The results of the searches were combined and validated using the Percolator module with filters set to high peptide identification confidence to achieve a false discovery rate (FDR) ≤1% for SEQUEST and ≤0.5% for Mascot. The proteomics data sets used in this study include proteome profiles from MCF-7 cell line. Proteins were identified from three 240 min gradient runs comprising 50 cells each. These results were compared to data sets comprising triplicate runs with samples comprising 100, 500, 1000, and 5000 MCF-7 cells.

#### Transcriptomics data

Gene expression data of MCF-7 cells (GEO accession: GSE21946) studied in Patacsil et al.'s work [32] were downloaded from Gene Expression Omnibus (GEO) [33]. The platform used in this study was Affymetrix Human Genome U133A 2.0 Array. Gene symbols were extracted from the platform data. The data from the Patacsil study was selected because it has both treated and control samples that show good reproducibility of results.

There were 8 samples in the array data and the expression levels of these 8 samples were averaged for each probe. For the genes with multiple probes, the highest average measurement was kept. The variation of gene expression between samples was minimal and we consider these data highly reproducible. For example, 97.7% of all transcripts showed the ratio of signals across 8 samples (max-min)/average at 0.2 (±10%) and 99.6% at 0.3 (±15%). *3) Gene sets/pathways data*

Canonical pathways used in this study were downloaded from MSigDB 3.1 [28]. There are 1452 gene sets included in this canonical pathways collection (CP collection). These gene sets represent well-described biological processes compiled by domain experts, which include gene sets derived mainly from BioCarta pathway database [34], KEGG pathway database [35], and Reactome pathway database [36]. These canonical pathways mainly include metabolic and signalling pathways that are shared by all cell types.

### Protein/gene pre-processing

#### cRAP proteins

The Common Repository of Adventitious Proteins [37] include proteins used in proteomics experiments, contaminants, or proteins used as quantitation standards. Proteins that were removed from proteomics data were: *ALBU_BOVIN, ALDOA_RABIT, CAH2_BOVIN, CAS1_BOVIN, CAS2_BOVIN, DHE3_BOVIN, LYSC_LYSEN, TRY1_BOVIN and TRYP_PIG*

#### Proteins without gene annotation

The gene symbols were extracted from the proteomics data report. Proteins without gene annotation were searched in UniProt [38,39] for their gene symbols, if available.

#### HGNC Gene Nomenclature

The gene names/symbols in proteomics data, transcriptomics data and gene sets in MSigDB are inconsistent in many cases. To solve this problem, all the approved gene symbols and their previous gene symbols/synonyms were downloaded from the HUGO Gene Nomenclature Committee - HGNC [40] on 8^th ^April, 2013. We screened all genes in above datasets and all gene names/symbols were mapped to the names approved by HUGO.

### Overlapping genes between proteomics and transcriptomics data

The genes names in the transcriptomics data were sorted in descending order of their RNA expression levels. The genes that were also detected in proteomics data were marked as 1, while the others were marked as 0. Sliding window of size 50 was applied here: the scores were added up for every consecutive 50 genes on the list. This method enables inspection of the overlaps between proteomics data and the transcriptomics data and find out protein content distribution relative to the transcript expression level.

### Gene set enrichment analysis

Hypergeometric distribution was applied to calculate the gene set enriched with proteins observed in 50-cell samples. For example, to investigate the gene set enrichment of a group of protein set PS in gene set A, probability will be calculated as:

$$P\left(X=k\right)=\frac{\left(\frac{K}{k}\right)\left(\frac{N-K}{n-k}\right)}{\left(\frac{N}{n}\right)}$$

where N is the number of genes in gene sets collections, K is the number of genes in gene set A, n is the number of proteins in PS, k is the number of proteins in PS that overlap with gene set A. The p-value of "if PS is enriched in A" will be calculated by summing up the probability from *P(X=k) *to *P(X=n)*.

Gene set enrichment analysis was done for each run of proteomics data from 50-cell samples and the gene set used was CP collections. Gene sets with p-value below 0.01 were kept for further analysis.

### Verification of predicted genes

Genes in mapped gene set were compared to the detected targets in each run and all detected targets in proteomics data as well.

## Results

### Proteins/genes detected in proteomics data

Mass spectrometry proteomics analysis was performed in experiments that used samples of 50-, 100-, 500-, 1500-, and 5000-cell sample sizes. Each sample was run in triplicate. The Common Repository of Adventitious Proteins (cRAP) collects proteins that are commonly found in proteomics experiments due to accidents or contamination of protein samples and was used to eliminate these proteins. After removing cRAP and duplicate proteins from the lists, a total of 5,032 proteins were detected from the proteomics data.

**Figure 2:**
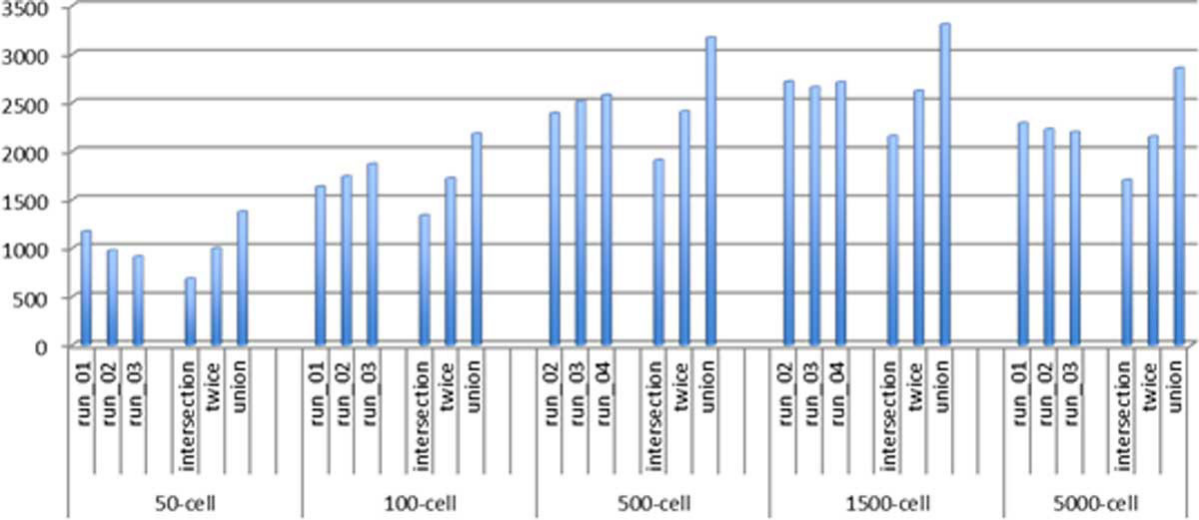
**Numbers of proteins identified in individual experiments and triplicate runs**. The "intersection" stands for proteins detected in all three runs, "twice" stands for proteins identified in at least two of all three runs, while "union" stands for proteins detected in any one run of all three runs.

The detected protein names were mapped to the approved gene symbols according to HGNC nomenclature (referred to as the approved gene symbols in the following text) [39], resulting in 4,957 identified and annotated proteins. The numbers of proteins identified in individual runs are shown in Figure 2. Larger samples yielded larger numbers of identified proteins, except for runs with 5,000-cell samples. This decline is the artefact of saturation of specific plot column [41] that was used this set of proteomics runs.

In three individual mass spectrometry runs with 50-cell samples, there were 1170, 972, and 909 proteins that could be mapped to the approved gene symbols. The numbers of proteins within the "intersection", "twice" (list of proteins identified in at least two of three runs), and "union" groups were 682, 995, and 1373 respectively. Assuming that the expressed proteome comprises approximately 10,000 expressed proteins [7], we can make a rough estimate of proteome coverage from small sample runs. The small sample runs (50-cell samples), therefore yielded approximately 1,000 proteins representing 10% of the expressed proteome, while total number of proteins identified in all runs (50-, 100-, 500-, 1500-, and 5000-cell samples) was close to 5,000 (4,957 identified and annotated proteins) representing approximately 50% of expressed proteome (Table 1).

**Table 1:** Enrichment of proteomics data in expression level-grouped transcriptomics data.

Threshold	Number of proteins transcripts	Number of mapped	% of proteins mapped to transcript data	% of all protein detected (within 4,957)
**≥2.8**	13,187	3,989	27.14%	80.47%
**≥6.0**	7,757	3,579	46.14%	72.20%
<6.0	5,430	410	7.55%	8.27%
**Absent from**	NA	968	NA	19.52% **transcript set**

RNA expression level 2.8 and 6.0 were used as thresholds.

### Genes and their expression level in transcriptomics data

**Figure 3:**
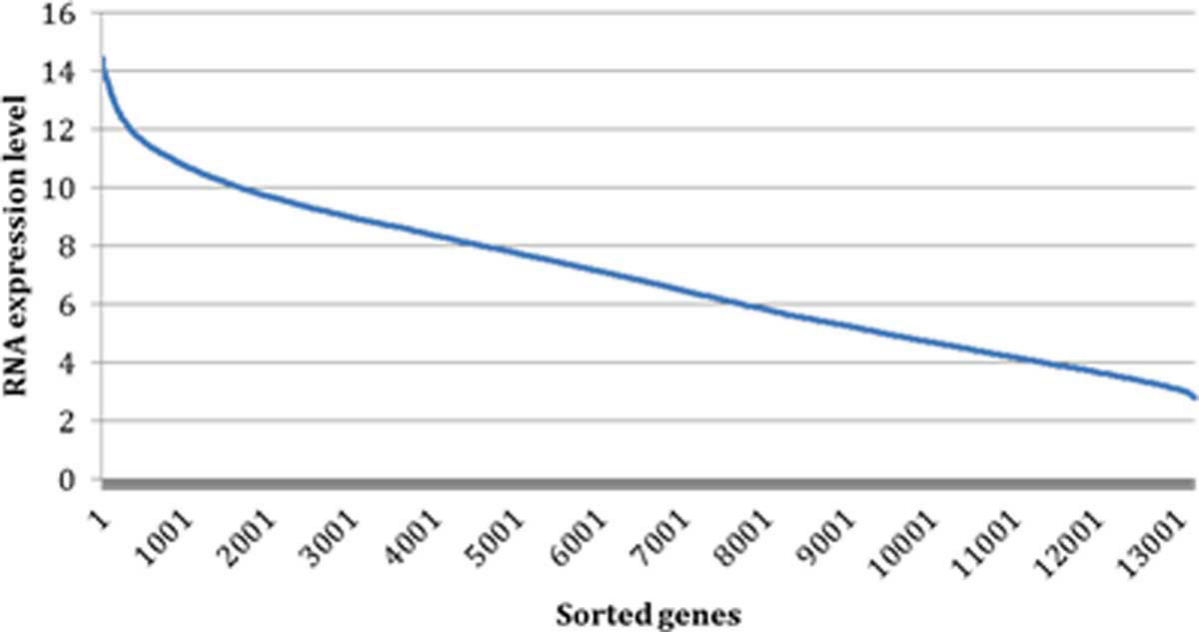
**The sorted RNA expression level in transcriptomics data**. In total, 13,187 genes were sorted from highest to lowest according to their respective highest RNA expression level.

Transcriptomics data from the Patacsil study [32] contained 13,187 unique transcripts that could be mapped to the approved gene symbols. These non-redundant transcripts were sorted from high to low expression ranging from 14.48 to 2.80 units (Figure 3). We selected 6.00 units as the tentative positive threshold.

### Mapping of proteomics data onto transcriptomics data

The analysis of the overlap between proteomics and transcriptomics data resulted in 3,989 proteins, representing 80.47% of the identified proteome. The remaining 19.53% of identified proteome was not available for transcript analysis (Table 1).

#### Cumulative analysis

**Figure 4:**
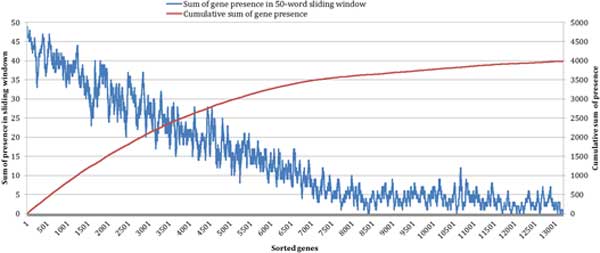
**Mapping of detected proteins in the transcriptomics data in MCF-7 samples**. The X-axis represents the number of transcripts sorted from the highest to the lowest expression level. The red line represents cumulative sum of identified proteins within the transcriptomics data. The blue line represents the number of proteins present as transcripts within the 50-member sliding window of transcript data.

From the cumulative result of protein presence in the sorted transcript list (Figure 4), it can be seen that the cumulative sum initially rises quickly and then plateaus as the level of RNA expression decreases.

#### Sliding window analysis

To assess the relationship of transcript expression and presence of expressed proteins we analysed the presence of expressed proteins in the sliding window of 50 transcripts sorted from the highest to the lowest expression level. Transcripts that had high expression level were more likely to have their corresponding protein expressed ranging from 98-100% (transcript expression level >14) to approximately 10% (transcript expression level <6) (Figure 4). The protein presence numbers within the 50-member transcript sliding window drops linearly from the highest expression transcripts to become stable after around ~7,500 transcripts in the sorted list. This corresponds well with the selected threshold for the positives of transcript expression (≥6 units with 7,757 individual transcripts were deemed positive).

### Enrichment of proteomics data in expression-level-grouped transcriptomics data

Given the results of concordance of protein and transcript expression shown in Figure 3, RNA expression level 6.0 was chosen as a threshold to cluster genes in transcriptomics data: higher expression group (genes with highest RNA expression level above or equal to 6.0) and lower expression group (genes with highest RNA expression level below 6.0).

The gene presence of proteomics data were further analysed in these two expression groups (Table 1). Proteomics data in genes with higher RNA expression level (greater than or equal to 6.0) is obviously more enriched than those in genes with lower RNA expression level.

### Mapping of proteins from small samples to canonical pathways (CP collections)

For each single run using a 50-cell sample, all proteins that could be mapped to the approved gene symbols were compared to all the gene sets in canonical pathway collections. Gene sets in the canonical pathways collection enriched with the expressed proteins detected by mass spectrometry were mapped, and all genes present in these gene sets were extracted. The detailed results are summarized in Table 2. The numbers of mapped gene sets are similar across three runs indicating that similar canonical pathways were deduced from protein expression results of individual runs. Among the 225, 227 and 221 gene sets mapped from "intersection" Runs 1, 2 and 3, 192 were identical, representing common canonical pathways shared between three runs. For the "twice" and "union" sets, larger number of canonical pathways were mapped resulting in a larger number of mapped genes.

**Table 2:** Gene sets mapping from CP collections.

	# proteins/genes detected	exist in CP^1^	# mapped gene sets	# genes in mapped gene sets
**Run 1 **	1,170	864	225	4,231
**Run 2 **	972	728	227	4,019
**Run 3 **	909	677	221	4,137
***Common pathways*** ***for 3 runs ***	*682 *	*536 *	*192 *	*3,828 *

**Intersection **	682	536	190	3,561
**Twice **	996	753	229	4,119
**Union **	1,373	980	244	4,273

"Gene sets" represent genes defined in CPs. The **number of genes represents the sum of different genes **across mapped CP.

^1 ^Not all proteins detected in 50-cell sample are present in the gene sets within the CP collection. The number of proteins found in the CP collections is shown here.

The number of proteins in the smallest set, comprising proteins that were detected in each of the three runs, was 682 constituting 58.5%, 70.2% and 75.0% of proteins detected in the Runs 1, 2, and 3. The number of gene sets identified from each small sample proteomic run is very similar, 221-227, and they are similar to the "twice" group. Intersection yields the smallest number of mapped pathways, 190, while "union" group yielded 244 gene sets. These sets are largely overlapping indicating the subsets of the same proteomes have been captured in each run.

### Verification of mapped genes derived from small sample

#### From mapped genes to proteomics data

**Figure 5:**
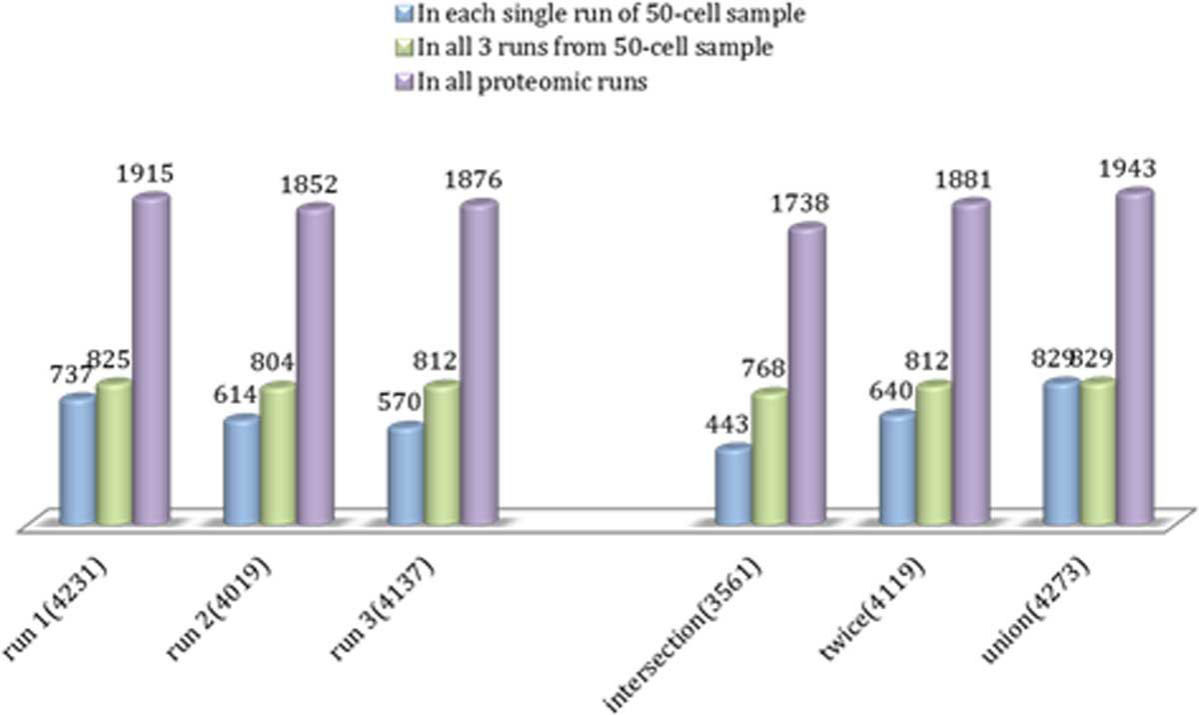
**The numbers of mapped genes (from enriched gene sets in canonical pathway collection) generated from 50-cell proteomics data**. The six groups of bars represent the result of each run in 50-cell sample, intersection of three runs, at least shown in two runs, and union of three runs. The numbers in brackets after group name are the numbers of mapped genes in enriched gene sets derived from detected targets in six groups. In each group, the bars represent the number of overlapping genes between mapped genes and 1) detected targets in respective group(blue bars), 2) detected targets in all three runs of 50-cell sample (green bars), and 3) detected targets in all proteomics runs, including 100-, 500-, 1500-, 5000-cells samples (purple bars).

For the targets derived from each single run of 50-cell proteomics samples, 17.42%, 15.28% and 13.78% (737 of 4,231 in Run 1, 614 of 4,019 in Run 2, and 570 of 4,137 in Run 3) were directly detected (Figure 5). Mapping the genes using union of the three runs, resulted in capturing of approximately 20% of expressed proteome. Approximately 45% of target genes identified by pathway analysis were present in the detected proteome. Assuming that the detected proteome represents 50% of actual proteome, we deduced that 90% of the proposed target genes identified from CPs (approximately 3,800 genes) were be expressed as protein products in the actual proteome. This hypothesis will need to be validated experimentally. We expect that the analysis of canonical pathways, therefore, will identify about 45% of the total proteome present in the samples. These canonical pathways mainly include metabolic and signalling pathways that are shared by all cell types. The remaining 55% of actual proteome will include proteins that do not have defined transcripts, or those that are defined by tissue-, process-, or disease-specific pathways.

#### From mapped genes to transcriptomics data

Approximately 83% of the mapped genes from CPs from any single run of 50-cell samples can be found in transcriptomics data (Table 3). Furthermore, of the proteins detected in any of the proteomic runs from our data, 80% can be found in transcriptomics data. The proportion of detected proteins that can be found in transcriptomic data, therefore, is similar for small and larger samples. However the overlap between the mapped genes and detected proteins is only 45% (e.g. 1915 mapped genes from run 1 were present in the set of all detected proteins, see Figure 5). Our results might be biased since mapped genes are members of the canonical pathways, while this bias does not exist in protein detection. Furthermore, transcriptome profiling was done in a different study and, although transcript and protein expression of MCF-7 cell line is expected to be relatively stable, our results are likely to capture the study-specific differences.

**Table 3:** The overlapping numbers of mapped genes from 50-cell sample, detected proteins in all proteomic runs to transcriptomics data.

		# mapped genes	Presence in transcriptomics data
**50-cell sample **	**Run 1** **Run 2** **Run 3** **Intersection **	4,231 4,019 4,137 3,561	3,519 (83.17%) 3,344 (83.20%) 3,437 (83.08%) 2,967 (83.32%)
	**Twice **	4,119	3,423 (83.10%)
	**Union **	4,273	3,560 (83.31%)

		**# detected proteins **	

**All proteomic runs **		4,957	3,989 (80.50%)

**Figure 6:**
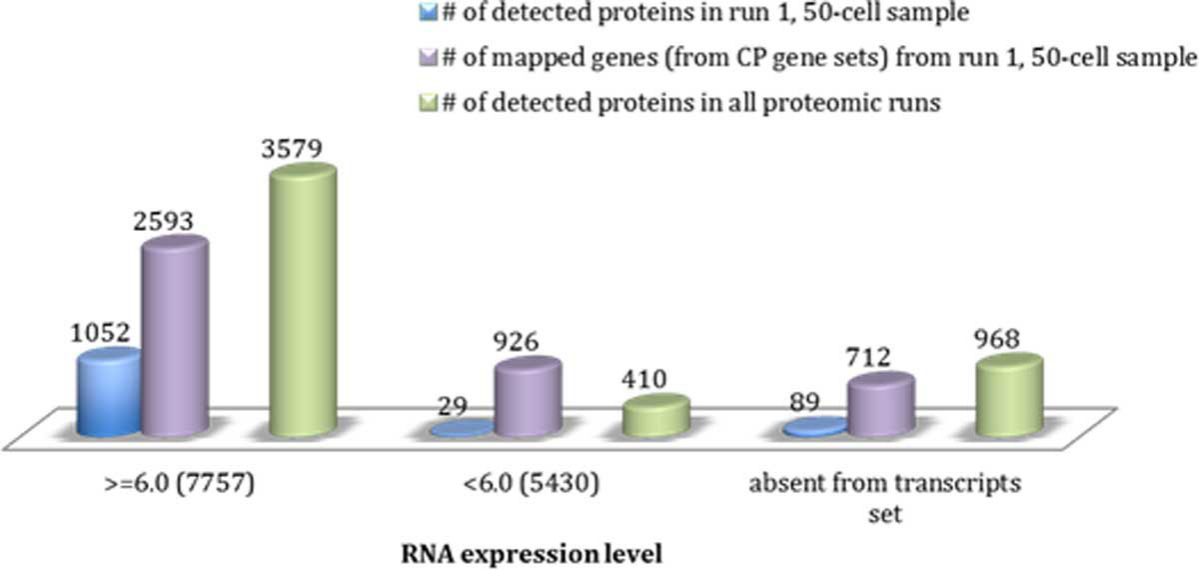
**The numbers of targets present in different RNA expression level groups**: **detected proteins in 50-cell run 1 (blue bars), mapped genes from 50-cell run 1 (purple bars), and detected proteins from all proteomic runs, including 100-, 500-, 1000-, 5000-cell samples (green bars)**. The overlap of the above targets in higher RNA expression group, lower RNA expression group, and absent from transcript set are shown separately from left to right. The numbers in brackets after RNA expression level groups represent the numbers of transcripts in each group.

The mapping data was further compared to the two RNA expression groups ≥6.0 and <6.0 (Table 1). Looking at data from run 1 in 50-cell sample for example, the number of proteins detected in run 1, gene expression data, and proteins detected in all proteomic runs were compared (Figure 6). Only 2.48% (29 proteins in 1170) that were detected in 50-cell run 1 have transcript expression of less than 6.0. Of all detected proteins, 8.27% (410 proteins in 4,957) have transcript expression of less than 6.0. This result is consistent with the sliding window analysis (see Figure 4) where approximately 10% (5 of 50) of proteins were shown to have low RNA expression (the horizontal part after first 7,500 transcripts). For mapped proteins in Run 1, this proportion is 21.89% (926 proteins from 4,231) indicating that some of the targets deduced from CPs, possibly 10% of the total, do not have protein product.

Alternatively, these differences may be attributed to differences in samples used in transcriptomic and proteomic experiments, but may also represent the products that are part of canonical pathways that do not have protein expression in the studied samples.

## Discussion and conclusions

Small sample analysis of expressed proteome is critical for many clinical samples since they represent points in time for disease progression in individual patients. We used the MCF-7 breast cancer cell line to benchmark the number of proteins that can be detected by using microscale proteomics and have developed strategies to increase the coverage of protein detection.

Proteomics data suffer from problems of coverage and consistency. The problem becomes worse as the sample size diminishes. In this study, pathway analysis has been confirmed as a useful method for improving protein identification in proteomics data. Approximately 1,000 proteins were detected in each small sample run followed by the identification of approximately 4,000 possible expressed protein targets. The proteomics data from larger samples experimentally validated approximately half of these probable targets in this study. Comparing these 4,000 possible targets to transcriptomics data, more than 80% of targets are highly likely to be present, especially enriched in the group of higher RNA expression. Our estimate is that only 10% of predicted proteome by canonical pathways may represent false positives. In addition, it appears that the predicted proteomes based on each individual run, intersection of proteins from three runs, or union of proteins from all three runs will produce very similar predicted proteomes. This indicates a remarkable robustness of the method reported in this study.

Naming conventions and nomenclature raise a problem when processed data are derived from multiple sources. It is also a problem when data are derived from a single source at different time points because of the changes and updates of gene and protein names. We have used the standardized symbols and have mapped proteomics, transcriptomics, and gene set (pathway) data to the common list of HUGO gene symbols. Approximately 20% of detected proteome could not be mapped to the HUGO gene symbols because these proteins either did not have corresponding gene symbols, the names were ambiguous and could not be resolved, or the products have been removed from the recent database update as obsolete or redundant.

Proteomics technology has improved and we can detect a significant proportion of the expressed proteome from small samples, such as 50 cells samples. However this detection initially amounts to only 10% of the estimated total expressed proteome. Knowledge-based approaches are needed to elucidate the likely presence of proteins that can be subsequently detected by targeted proteomics. These KB-approaches include analysis of pathways and combination of gene expression and protein expression data. Using meta-analysis, we have shown that most of the proteins, perhaps 90%, identified as members of canonical pathways - pathways common for all cell types - are likely to be expressed as proteins. These proteins represent approximately 50% of the expressed proteome. The remaining proteins can be elucidated by the analysis of tissue-, organ-, process-, or disease-specific pathways. Furthermore, targets that are represented by highly expressed transcripts are more likely to be expressed as proteins (98-100% for transcripts that show highest expression levels). Approximately 10% of transcripts that show low or no expression have their proteins expressed, as detected in the proteomics runs. This may include a number of false positives due to different sources used in this study for the analysis of transcriptome and proteome, but it is highly likely that the majority of expressed proteins are real.

In summary, proteomics detection of protein expression from small samples can be enriched by pathway analysis followed by targeted proteomics. Furthermore, gene expression data can be used for prioritization of potential targets for deep proteomics screening. This study has provided benchmark results that will facilitate proteogenomics studies for detecting expressed proteomes from samples comprising small numbers of cells.

## Authors' contributions

VB, ARI, and BK designed the study. SKM prepared and provided MCF-7 samples for proteomic analysis. ARI and SL performed proteomics experiments and the initial data analysis and provided the resulting data sets for knowledge-based approaches. JS, GLZ, and VB collected and standardized data and performed the analysis. DF and FL provided the conceptual framework for the study and critically read the article. All authors have read the article and contributed to the main text.

The authors declare that they have no competing interests.

## Declaration

The cost of this publication was funded by Vladimir Brusic.

This article has been published as part of *BMC Genomics *Volume 15 Supplement 9, 2014: Thirteenth International Conference on Bioinformatics (InCoB2014): Computational Biology. The full contents of the supplement are available online at http://www.biomedcentral.com/bmcgenomics/supplements/15/S9.
